# Radial head arthroplasty restores axial construct stiffness in a Monteggia-like injury model: a biomechanical study

**DOI:** 10.1007/s00402-026-06515-w

**Published:** 2026-10-28

**Authors:** Marc Maximilian Weber, Jan P. Hockmann, Till A. Rosenkranz, Tim Leschinger, Lars Peter Müller, Valentin Rausch

**Affiliations:** Center for Orthopedic and Trauma Surgery, University Hospital Cologne, Cologne, Germany

**Keywords:** Monteggia injury, Elbow trauma, Olecranon fracture, Radial head fracture, Elbow dislocation, Ulna non-union

## Abstract

**Introduction:**

Monteggia-like lesions are rare but complex elbow injuries involving fractures of both the proximal ulna and the radial head. While the influence of ulnar alignment and soft tissue integrity on radial head stability has been extensively studied, the effect of radial head integrity on the stability of ulnar fixation remains poorly understood. Given the high rates of nonunion and revision surgery after proximal ulna fractures, improved understanding of forearm biomechanics in these combined injuries is highly clinically relevant as it influences the surgical strategy in primary care of these injuries. Accordingly, this study aimed to evaluate the biomechanical impact of radial head resection and radial head arthroplasty on forearm stiffness in a simulated Monteggia-like injury model. We hypothesized that radial head resection would reduce construct stiffness compared with both the native state and radial head arthroplasty, and that arthroplasty would partially restore stiffness toward native levels

**Materials and methods:**

Eight fresh-frozen human cadaveric elbow specimens were biomechanically tested under axial loading. After baseline testing in the native state, a standardized segmental comminution model was created and stabilized using locking plate osteosynthesis. Sequential testing was then performed after radial head resection and after implantation of a modular radial head prosthesis. Specimens were loaded cyclically up to 450 N for 1,000 cycles with the elbow positioned at 30° of flexion. Construct stiffness was calculated and compared between testing conditions.

**Results:**

Mean construct stiffness in the native state was 49.3 ± 4.29 N/mm and decreased significantly after creation and fixation of the ulnar defect (36.05 ± 6.71 N/mm). Radial head resection resulted in a further significant reduction in stiffness (25.83 ± 6.65 N/mm). Following radial head arthroplasty, stiffness increased to 33.8 ± 7.89 N/mm, representing a significant improvement compared with the resection state (p **≤ **0.0026). No significant difference was observed between stiffness after prosthesis implantation and the condition with intact radial head and stabilized ulna.

**Conclusion:**

Radial head integrity significantly contributes to forearm stiffness in Monteggia-like lesions. Radial head arthroplasty partially restores biomechanical stability after resection and may alter load distribution in a manner that could be relevant for fixation durability. These findings support the need to address the radial column to optimize stability in complex elbow injuries.

**Level of evidence:**

Biomechanical study

## Introduction

Monteggia injuries were first described by Giovanni Battista Monteggia in 1814 as a combination of a proximal ulna fracture and dislocation of the radial head. Since then, several variants of this injury pattern have been recognized, including Monteggia-like lesions, which involve a fracture of the proximal ulna combined with a fracture-dislocation of the radial head. These injuries are rare, accounting for approximately 1–5% of elbow fractures, but remain challenging to treat due to their complex osseous and ligamentous injury patterns [1–5].

Operative treatment is generally required, with locking plate osteosynthesis representing the current standard for fixation of proximal ulna fractures [6–8]. In contrast, the optimal management of the radial head remains controversial. Available options include open reduction and internal fixation (ORIF), radial head arthroplasty (RHA), or radial head excision. While reconstruction of the radial head is preferred whenever feasible, highly comminuted fractures often require prosthetic replacement or resection [9–12].

Previous studies have primarily focused on the influence of ulnar alignment, forearm position, and soft-tissue integrity on the stability of the radial head in Monteggia injuries [13–15]. However, the reciprocal biomechanical relationship—namely the influence of radial head integrity on the stability of ulnar fixation—has received considerably less attention. This question is clinically relevant, as proximal ulna fractures in Monteggia-like lesions are associated with substantial rates of nonunion and early revision surgery [16].

Therefore, the aim of the present study was to investigate the biomechanical impact of radial head resection and radial head arthroplasty on forearm stiffness in a simulated Monteggia-like injury model.

## Materials and methods

### Specimen preparation

Sample size was determined based on data from comparable biomechanical cadaveric studies reporting construct stiffness values in elbow fracture models. Assuming a clinically relevant mean difference of 8 N/mm between testing conditions with an estimated standard deviation of 6.6 N/mm and a within-subject correlation of *r* = 0.6, a minimum of 6 specimens were calculated to achieve 80% power at a two-sided significance level of α = 0.05. Eight specimens were included to account for potential specimen loss and to provide an additional safety margin.

The specimens were stored at −20 °C and thawed at room temperature 16–18 h before dissection and biomechanical testing. Fluoroscopic and clinical examinations were performed to exclude specimens with osteoarthritis or signs of previous surgery and trauma.

At the mid-diaphyseal level of the humeral shaft, the elbow and forearm were transected and separated from the shoulder and proximal humerus. Proximal soft tissues were removed, preserving only the humeral shaft, which was subsequently embedded in PMMA. After native biomechanical testing of the intact specimen (protocol described below), further preparation and sequential testing were performed.

The proximal ulna was exposed through a posterior approach. Immediately distal to the base of the coronoid process, a 5-mm-wide segmental defect was created in the ulnar shaft to simulate a multifragmentary fracture. The defect was bridged using an olecranon plate (Variax System; Stryker, Kalamazoo, MI, USA), fixed proximally and distally with one cortical screw and two locking screws on each side. A second biomechanical measurement was then obtained.

Following the second measurement, the radial head was resected with preservation of the lateral ligamentous complex, and testing was repeated. Subsequently, a radial head prosthesis (Evolve; Stryker, Kalamazoo, MI, USA) was implanted. Prosthesis size and height were selected based on the dimensions of the resected radial head (Table 1). Correct implant positioning was confirmed fluoroscopically, after which the final stiffness measurement was conducted.

**Table 1:** Characteristics of radial head prosthesis implantation, including prosthesis size, stem diameter, and implant height selected for each specimen

Number	Radial head		Stem	
	diameter (mm)	height (mm)	diameter (mm)	height (mm)
1	24	4	6,5	4
2	22	0	6,5	2
3	22	2	7,5	2
4	22	0	6,5	2
5	24	2	8,5	2
6	24	0	7,5	2
7	22	2	5,5	4
8	20	2	5,5	2

### Biomechanical testing set-up

**Fig. 1:**
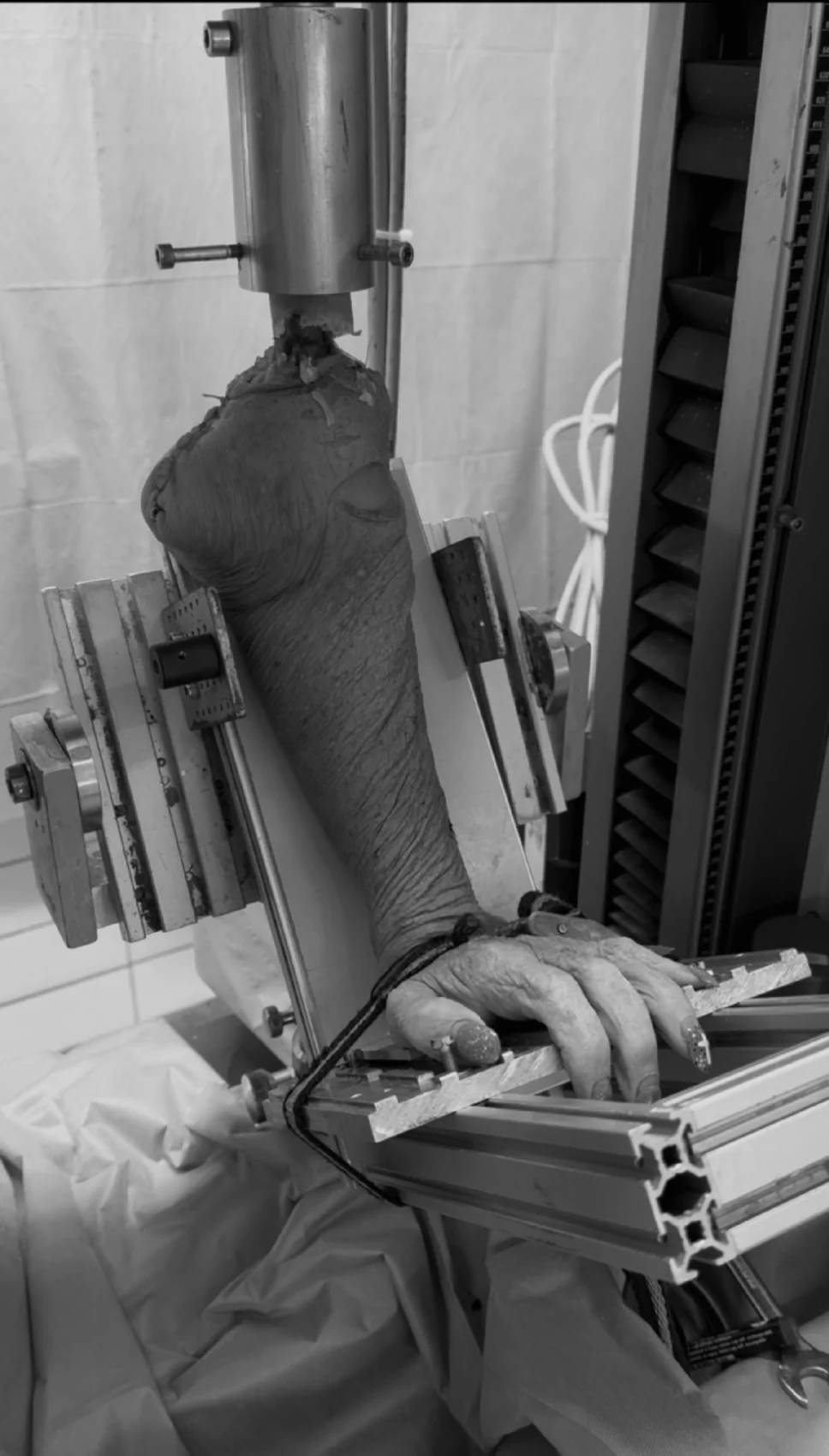
Biomechanical testing set up: The PMMA-embedded humeral shaft was fixed to the crosshead of the testing machine. A custom-made holding device guided the elbow at 30° of flexion, enabling distal support of the hand and controlled load transmission through the forearm

After embedding of the humeral shaft, the specimen was mounted vertically in a servohydraulic testing machine (Zo10; Zwick/Roell, Ulm, Germany). The forearm was positioned in pronation with the elbow flexed to 30° using a custom-made fixation device. The elbow was positioned at 30° of flexion to simulate a physiologically relevant loading condition. This position represents the transition from bony to ligamentous stabilization of the elbow joint and reflects a commonly assumed posture during daily activities, as full extension is rarely adopted under load. Furthermore, as radiocapitellar contact areas have been shown to be independent of elbow flexion angle, the chosen position can be considered representative across the functional arc of motion [17, 18]. The hand was secured to a rigid plate with a strap, maintaining the wrist in 90° of dorsiflexion. Downward displacement of the mobile crosshead generated compressive loading (Fig. 1).

A preload of 5 N was applied, followed by cyclic axial loading up to 450 N for 1,000 cycles at a displacement rate of 1 mm/s. The number of 1,000 loading cycles was chosen in accordance with previously published biomechanical elbow studies employing cyclic axial loading protocols [19]. A maximum vertical displacement of 20 mm was predefined as the failure criterion, at which point testing was terminated. Applied load (N) and axial displacement (mm) were continuously recorded. Construct stiffness was calculated as the ratio of peak axial load to peak axial displacement recorded during each test cycle (stiffness = Fmax/dL (Fmax)), representing the secant stiffness at maximum load.

Throughout all testing procedures, specimens were kept moist using saline-soaked towels. The testing sequence was identical for all specimens.

### Statistical analysis

Microsoft^®^ Excel^®^ 2011 for Mac and GraphPad PRISM 10 (Graphpad Software, Inc) were used for data collection, data visualization and statistical analyses.

Normal distribution of all variables was confirmed using the Shapiro-Wilk test. As all testing conditions met the assumption of normality, paired Student’s t-tests were applied for all comparisons. Results are presented as mean ± standard deviation. A *P* value of < 0.05 was considered statistically significant.

## Results

### Specimen characteristics

Eight fresh-frozen human elbow specimens were included in the biomechanical analysis. The mean age of the donors was 78 ± 11 years (range, 53–91 years). The specimen cohort comprised seven female and one male donor. Four left-sided and four right-sided elbows were tested. All specimens completed the full testing protocol without exclusion (Table 2).

**Table 2:** Specimen characteristics and biomechanical results

Number	Stiffness (*N*/mm) native	Stiffness (*N*/mm) Ulna fracture	Stiffness (*N*/mm) Radial head resected	Stiffness (*N*/mm) Radial head prosthesis	Age	Sex
LS07L	50.94	28.19	15.31	19.99	76	female
LS16R	52.04	34.067	19.69	27.97	86	female
LS18L	47.95	33.04	26.21	30.01	53	female
LS19L	44.1	41.6	29.98	35.77	82	female
LS20	45.93	41.2	36.63	39.89	75	male
LS21R	48.81	38.03	25.16	41.27	77	female
Z45R	47.09	26.67	23.31	40.5	84	female
Z49L	57.5	45.6	30.32	35.02	91	female
Mean	49.3	36.0	25.8	33.8	78.0	

Donor age and sex are summarized for each specimen, along with mean construct stiffness values (N/mm) for all testing conditions

### Biomechanical results

All eight specimens completed the full testing protocol without reaching the predefined failure criterion of 20 mm axial displacement. Peak forces remained stable throughout cyclic loading, with Fmax values consistently approximating the target load of 450 N, indicating the absence of relevant fatigue or progressive deformation during testing. Mean vertical stiffness values for each testing condition are summarized in Table 2.

Native testing demonstrated a mean stiffness of 49.3 ± 4.29 N/mm. Following creation of the proximal ulnar bone defect and fixation with an olecranon plate, stiffness decreased to 36.05 ± 6.71 N/mm. Subsequent radial head resection resulted in a further reduction in stiffness to 25.83 ± 6.65 N/mm. After implantation of a radial head prosthesis, stiffness increased to 33.8 ± 7.89 N/mm.

**Fig. 2:**
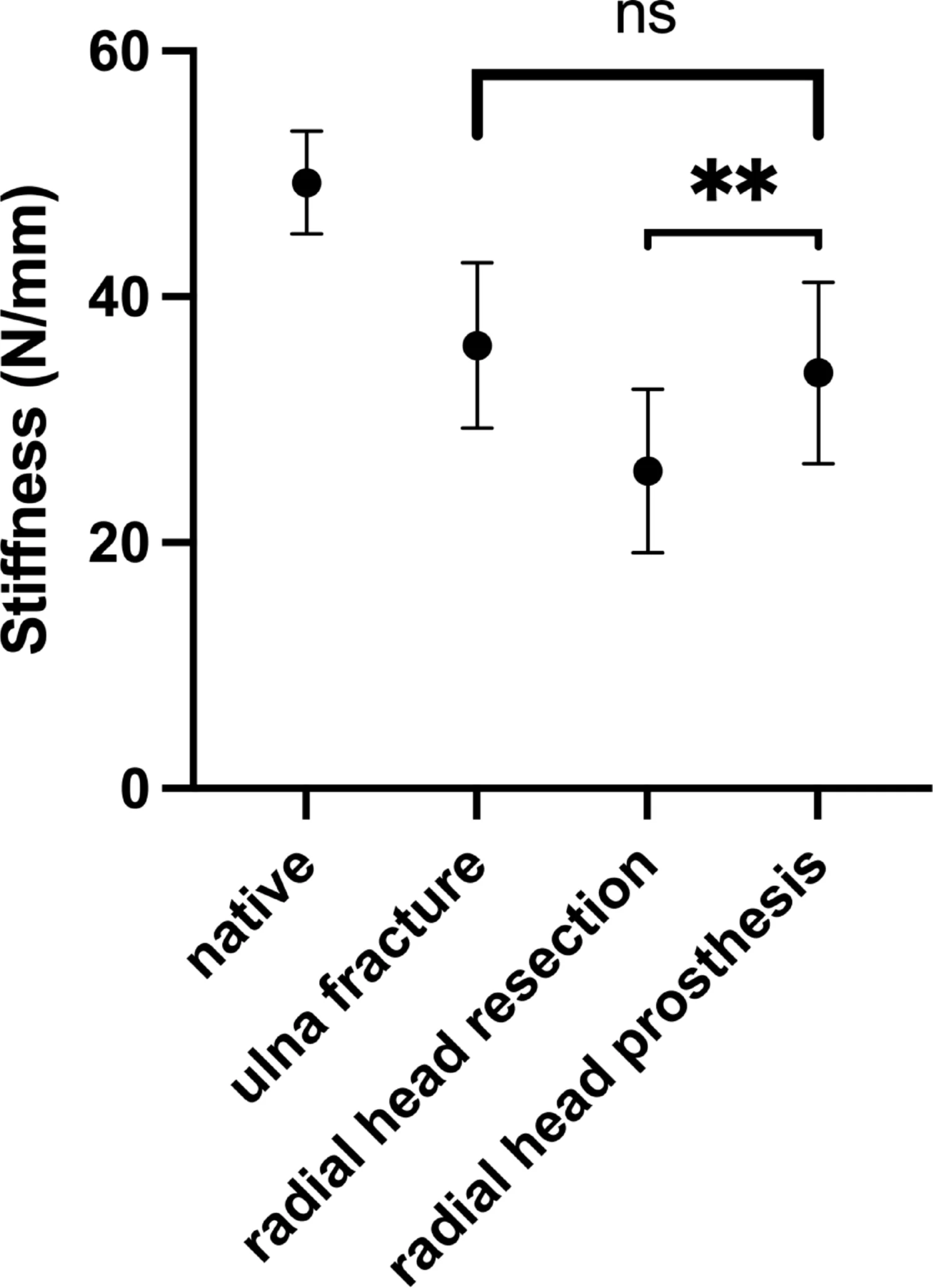
Mean construct stiffness (N/mm) under axial loading for the different testing conditions. Stiffness decreased after ulnar defect creation and further after radial head resection, while implantation of a radial head prosthesis partially restored stiffness. Error bars indicate standard deviation

Stiffness decreased significantly following creation of the ulnar defect (*p* = 0.001) and decreased further after radial head resection (*p* < 0.001). Implantation of a radial head prosthesis resulted in a significant increase in stiffness compared with the resection state (*p* = 0.005). Importantly, no significant difference was observed between stiffness after prosthesis implantation and the condition with an intact native radial head and stabilized ulna (*p* = 0.440), suggesting that arthroplasty restores construct stiffness to a level comparable to that achieved with preservation of the native radial head (Fig. 2).

## Discussion

The most important finding of the present study is that radial head integrity substantially contributes to axial construct stiffness in a simulated Monteggia-like injury model. While radial head resection resulted in a marked reduction of construct stiffness, implantation of a radial head prosthesis partially restored stiffness to levels comparable to those observed after stabilization of the ulna with an intact native radial head. These findings highlight the biomechanical importance of the radial column in the load transmission of the forearm and suggest that restoration of radial head integrity may contribute to improved stability of ulnar fixation constructs.

From a biomechanical perspective, the radial head plays a key role in axial load sharing between the radius and ulna. Loss of the radial head reduces the capacity of the radial column to transmit load, thereby increasing the proportion of axial forces transferred through the ulna. In the presence of a proximal ulna fracture stabilized with plate osteosynthesis, this altered load distribution may increase mechanical stress at the bone–implant interface and potentially compromise fixation stability. Restoration of the radial column through radial head arthroplasty appears to partially re-establish physiological load transmission and thereby improve construct stiffness.

Previous biomechanical studies have primarily focused on the influence of ulnar alignment, forearm rotation, and soft-tissue stabilizers on radial head stability in Monteggia injuries. In particular, recent work by Vannitamby et al. demonstrated that ulnar angulation, forearm position, and ligament reconstruction significantly influence radial head stability in anterior Monteggia fractures [14, 15, 20]. In contrast, comparatively little attention has been directed toward the reciprocal relationship, namely the influence of radial head integrity on the mechanical behavior of the fractured ulna. The findings of the present study suggest that radial head stability may also contribute to ulnar construct stability and therefore should be considered as part of a comprehensive biomechanical treatment concept.

The stabilizing effect of radial head arthroplasty observed in this study is consistent with previous biomechanical investigations demonstrating that radial head replacement can restore elbow stability after radial head resection [13]. However, most of these studies evaluated isolated radial head pathology. The present study extends this concept to combined injuries involving both the radial and ulnar columns of the forearm, thereby providing additional insight into the biomechanical interplay between these structures in Monteggia-like lesions.

Potential adverse effects of radial head arthroplasty must nevertheless be considered. Oversizing of radial head prostheses has been associated with increased radiocapitellar contact pressures and may contribute to the development of radiocapitellar arthrosis or altered joint mechanics [21, 22] Accurate prosthesis sizing and restoration of native radial length therefore remain critical to avoid iatrogenic joint overload.

From the perspective of ulnar fixation, advances in plating techniques have also aimed to improve construct stability. For example, “mother-and-baby plate” constructs have been proposed to enhance fixation strength in proximal ulna fractures [7]. It is conceivable that increased rigidity of ulnar fixation constructs may reduce the relative biomechanical contribution of the radial head to overall forearm stability. Whether improvements in ulnar fixation techniques diminish the stabilizing effect of the radial column remains unclear and warrants further investigation.

Clinical studies addressing radial head management in Monteggia-like lesions have reported variable results. Early investigations raised concerns regarding complication rates associated with radial head arthroplasty in this setting [23]. Subsequent studies emphasized that open reduction and internal fixation should be preferred whenever stable reconstruction of the radial head can be achieved, whereas arthroplasty may provide advantages in highly comminuted fractures [24]. More recent data, however, suggest that outcomes after radial head arthroplasty may depend strongly on surgical expertise and implant design. Contemporary studies have reported lower revision rates and favorable long-term outcomes, particularly with modern implant concepts and intentionally loose-fit prosthetic designs [25, 26].

Importantly, insufficient radial head fixation or radial head resection may contribute to early failure and the need for revision surgery following treatment of Monteggia fractures [12, 16] These observations support the concept that inadequate restoration of radial head stability may compromise overall elbow and forearm stability and thereby negatively influence fracture healing and fixation durability.

Taken together, the results of the present study emphasize the biomechanical interdependence of the radial and ulnar columns in Monteggia-like injuries. Although reconstruction of the radial head should be preferred when feasible, radial head arthroplasty may represent a reasonable option when anatomical reconstruction cannot be achieved. Ensuring adequate restoration of the radial column may help optimize load distribution within the forearm and potentially improve the mechanical environment for ulnar fracture healing.

This study has several limitations. First, it represents a cadaveric biomechanical model and therefore cannot fully reproduce the complex biological and biomechanical conditions of Monteggia-like injuries in vivo. In particular, dynamic muscular forces and neuromuscular control were not simulated. Second, only axial loading conditions were evaluated, whereas elbow stability in clinical scenarios is also influenced by rotational and varus–valgus forces. Third, the injury model did not include ligamentous injuries or proximal radioulnar joint instability, which are commonly present in Monteggia-like lesions. Finally, the number of specimens was limited and the donor population was relatively elderly. Despite these limitations, the standardized experimental setup allowed controlled assessment of the biomechanical contribution of radial head integrity to forearm construct stiffness.

## Conclusion

The present study suggests that radial head integrity plays an important role in restoring forearm stiffness and may contribute to the biomechanical stability of ulnar fixation in Monteggia-like injuries. These findings support a treatment concept that considers the reciprocal interaction between the radial head and the ulna and highlights the importance of addressing both structures during the initial surgical intervention.

## Data Availability

The datasets generated and analysed during the current study are available from the corresponding author on reasonable request.
